# Prediction of Distal Femoral and Posterior Articular Surfaces in Total Knee Arthroplasty With Severe Bone Defects Using Computed Tomography-Based Templating Software

**DOI:** 10.7759/cureus.61546

**Published:** 2024-06-02

**Authors:** Masashi Hirakawa, Masashi Miyazaki, Miyuki Sato, Nobuhiro Kaku

**Affiliations:** 1 Orthopaedics, Oita University, Yufu, JPN; 2 Orthopaedic Surgery, Miyuki Clinic, Oita, JPN; 3 Orthopaedic Surgery, Oita University, Yufu, JPN

**Keywords:** knee replacement surgery, femoral width, joint line, computed tomography (ct ), total knee arthroplasty (tka)

## Abstract

Introduction

Optimal repair of the joint line (JL) in total knee arthroplasty (TKA) is critical for knee joint motion reconstruction and ligament balance. Identification of JL may be difficult, particularly in revision or primary cases of severe femoral condylar bone loss. We aimed to define the relationship between the epicondyles and the articular surface (AS) of the femur using computed tomography-based three-dimensional digital templating software.

Methods

The study included 127 knees with osteoarthritis of the knee below grade 2 on the Kellgren-Lawrence index. A perpendicular line was drawn from the medial and lateral femoral epicondylar processes to the most distal point of the AS, and the distance was measured in the axial and coronal planes. Femoral width was measured as the distance between the medial and lateral epicondyles. All distances described above were converted to a ratio by division with femoral width.

Results

On the axial plane, the distance from epicondyles to the posterior ASs was 29.4 ± 2.2 mm medially and 21.3 ± 2.1 mm laterally. The width of the distal femur was 75.2 ± 4.2 mm. On the coronal plane, the distances from epicondyles to the distal ASs were 25.2 ± 2.9 mm on the medial side and 21.3 ± 2.5 mm on the lateral side. The ratio of the distance from epicondyles to the distal and posterior ASs divided by the width of the femur was 0.39 ± 0.02, 0.28 ± 0.03, 0.34 ± 0.03, and 0.28 ± 0.03.

Conclusions

The distance from the epicondyles to the distal and posterior JLs correlates with the distal femur width. These findings may be useful in determining an appropriate JL.

## Introduction

Total knee arthroplasty (TKA) is effective in relieving pain and restoring knee joint function in patients suffering from end-stage osteoarthritis and rheumatoid arthritis of the knee [1]. The average rate of TKA, including primary and revision, is 175 per 100,000 population, a rate that has increased significantly [2]. However, 11-19% of primary TKA patients are dissatisfied with the surgical outcome [3].

Joint line (JL) restoration in TKA is critical for knee kinematics and ligament balance. JL displacement has a significant negative impact on patellar kinematics and knee stability, and an elevation of the JL at the time of primary TKA has been proven to be associated with poor clinical outcomes [4-9]. Furthermore, JL is often elevated after revision total knee arthroplasty (rTKA), leading to a lower clinical assessment score. However, restoration of the JL during rTKA surgery yields significantly better results than leaving >5 mm without restoration [6].

In primary TKA or rTKA with severe deformities, identification of the JL may be difficult due to bone loss surrounding the femoral and tibial components. In many cases, the level of the JL is confirmed by measuring the absolute distance from the bony landmarks to the tangential line of the JL [10,11]. The most commonly used bone landmark is the femoral epicondyle, which can also be used for the rotation and proximal/distal positioning of the femoral component. We hypothesized that measurement of the articular surface (AS) from bony landmarks in a knee with minimal deformity could predict its location in cases wherein the AS is difficult to identify due to severe bone defects. Three-dimensional (3D) image-matching software can be used to accurately assess the relationship between the bony landmarks and ASs using computed tomography (CT) images. This study aimed to determine the relationship between the femoral epicondyle and knee AS using CT-based 3D digital template software to help reconstruct the AS of knees with advanced bone loss.

## Materials and methods

Patient population

The protocol for the present study was approved by the Institutional Review Board of Oita University Hospital (approval number: 1850; June 16, 2020). Since this study was retrospective and noninvasive, informed consent was obtained in the form of opt-out.

Anteroposterior and lateral radiographs and CT images of 88 patients (nine males and 79 females) and 127 knees (13 males and 114 females) with varus knee osteoarthritis were analyzed. The mean age of the subjects was 76.1 ± 5.6 years (range: 52-88 years) and Kellgren-Lawrence classification grade (K-L grade) was grade 2 or less. Patients with a history of fracture, knee surgery other than arthroscopy, or valgus knee deformity were excluded.

Analysis methods

**Figure 1:**
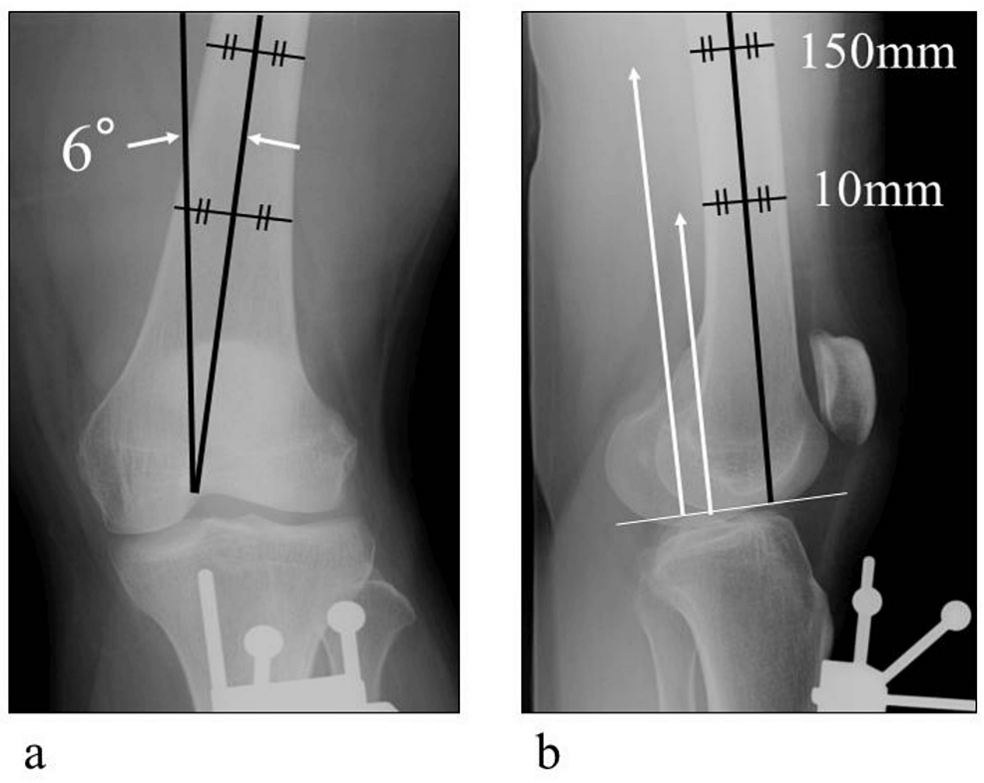
Definition of the radiographical axis. (a) The femoral mechanical axis was defined as the angle of 6° valgus to the femoral anatomical axis. (b) The femoral anatomical axis was defined as the line at the midpoint of the femoral width at 100 mm and 150 mm proximal to the distal joint line.

Anteroposterior and lateral radiographs were obtained, and evaluations were performed using Athena Knee 3D image-matching software (Soft Cube Co, Ltd, Osaka, Japan) [12]. A 3D marker was attached to the surface of the patient’s lower leg, and the silhouettes of the marker on the images were used to couple the two radiographic images in three dimensions. Subsequently, the CT images were matched with the coupled radiographic images. The femoral anatomic axis was defined as the line connecting the midpoints of the femoral width at 100 mm and 150 mm proximal to the distal JL. The femoral mechanical axis was defined as the angle of 6° valgus to the femoral anatomical axis (Figure 1). The axial plane is defined as the vertical plane of the femoral mechanical axis. The prominences of the medial femoral epicondyle (ME) and lateral femoral epicondyle (LE) were determined on the axial plane. The medial femoral epicondyle has two reference points: the prominence and the sulcus. As the prominence was easier to recognize than the sulcus of the MEs in all cases, the former was used as the reference point [13].

**Figure 2:**
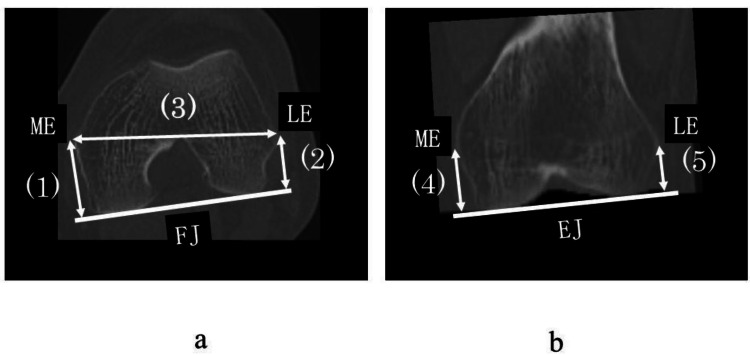
Measurement of parts of the distal femur. (a) The axial plane is defined as the vertical plane of the femoral mechanical axis. (1) ME-FJ: distance from the ME to the posterior AS in the flexed position. (2) LE-FJ: distance from the LE to the posterior AS in the flexed position. (3) ML: the width of the distal femur was measured as the distance between the ME and LE. (b) The coronal plane was defined as the plane containing the clinical transepicondylar axis (c-TEA) and the horizontal plane of the femoral mechanical axis. (4) ME-EJ: length from the ME to the distal AS in an extended position. (5) LE-EJ: distance from the LE to the distal AS in an extended position. JL: joint line; ME: medial femoral epicondyle; FJ: the JL in the flexed position; AS: articular surface; LE: lateral femoral epicondyle; EJ: JL in the extended position.

A perpendicular line was drawn from the ME and LE to the posterior condylar line, defined as the JL in the flexed position (FJ), and their distances were measured (Figure 2A). The coronal plane contained the clinical transepicondylar axis and the horizontal plane of the femoral mechanical axis. Perpendicular lines were dropped from the prominence of the ME and LE to the distal articular line, defined as the JL in the extended position (EJ) (Figure 2B). The following parameters were measured using the Athena imaging software: (1) ME-FJ: distance from the ME to the posterior AS in the flexed position; (2) LE-FJ: distance from the LE to the posterior AS in the flexed position; (3) ML: the width of the distal femur was measured as the distance between the ME and LE; (4) ME-EJ: length from the ME to the distal AS in an extended position; (5) LE-EJ: distance from the LE to the distal AS in an extended position. Each of the distances described above was converted into a ratio by division with the width of the distal femur.

Statistical analysis

Intra- and inter-observer measurement reliability was analyzed using the intra-class correlation coefficient (ICC). Means and standard deviations (SD) were calculated for descriptive analysis. Pearson’s correlation coefficient was used to evaluate the correlation between parameters. Statistical significance was set at P < 0.05. All statistical analyses were performed with SPSS version 18.0.0 software (SPSS, Inc., Chicago, IL). The post hoc power analysis was performed using G*Power 3.1.9.7. Assuming a two-tailed test for the population correlation coefficient with an expected correlation coefficient of 0.3, a significance level of 5%, and 127 cases, the post hoc power was calculated to be 94.0%.

## Results

Intra-observer and inter-observer variability

All ICC values were >0.94, indicating very high intra- and inter-observer reliabilities.

Measured distances and ratios

Mean ME-FJ was 29.4 ± 2.2 mm (95% CI: 29.0 to 29.7), mean LE-FJ was 21.2 ± 2.3 mm (95% CI: 20.8 to 21.6), ML was 75.1 ± 4.2 mm (95% CI: 74.4 to 75.9), mean ME-EJ was 25.1 ± 2.8 mm (95% CI: 24.6 to 25.6), mean LE-EJ was 21.3 ± 2.5 mm (95% CI: 20.9 to 21.8), the ratio between ML and ME-FJ was 0.39 ± 0.02, the ratio between ML and LE-FJ was 0.28 ± 0.03, the ratio between ML and ME-EJ was 0.33 ± 0.04, and the ratio between ML and LE-EJ was 0.28 ± 0.03 for the lateral side (Table 1).

**Table 1:** The mean length from each prominence of the epicondyle to the AS and the ratio of length divided by ML. ME-FJ: distance from the ME to the posterior AS in a flexed position; LE-FJ: distance from the LE to the posterior AS in a flexed position; ME-EJ: length from the ME to the distal AS in an extended position; LE-EJ: distance from the LE to the distal AS in an extended position; ML: width of the distal femur was measured as the distance between the ME and LE; JL: joint line; ME: medial femoral epicondyle; FJ: the JL in the flexed position; AS: articular surface; LE: lateral femoral epicondyle; EJ: the JL in the extended position.

Parameters	Length (mm)	A ratio by dividing by ML
ME-FJ	29.4 ± 2.2	0.39 ± 0.02
LE-FJ	21.2 ± 2.3	0.28 ± 0.03
ME-EJ	25.1 ± 2.8	0.33 ± 0.04
LE-EJ	21.3 ± 2.5	0.28 ± 0.03
ML	75.1 ± 4.2	N/A

Pearson correlation coefficients

**Figure 3:**
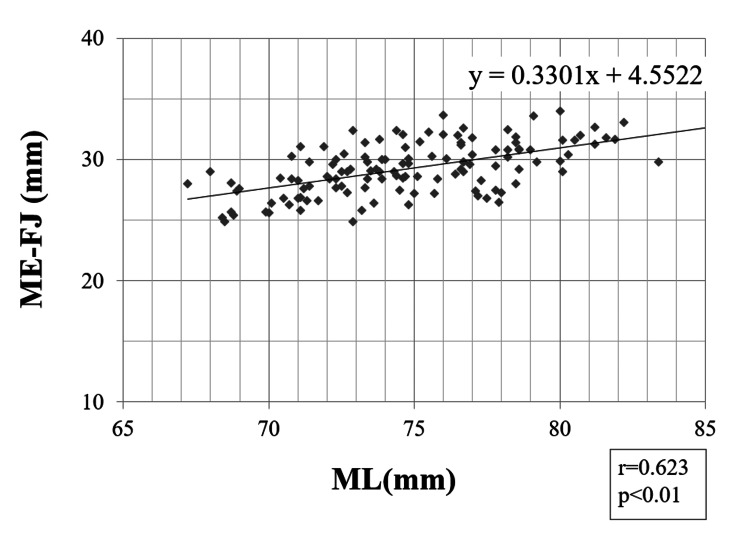
Correlation between ME-FJ and ML. ME-FJ: distance from the ME to the posterior AS in the flexed position; ML: the width of the distal femur was measured as the distance between the ME and LE; ME: medial femoral epicondyle; FJ: the JL in the flexed position; AS: articular surface; LE: lateral femoral epicondyle.

**Figure 4:**
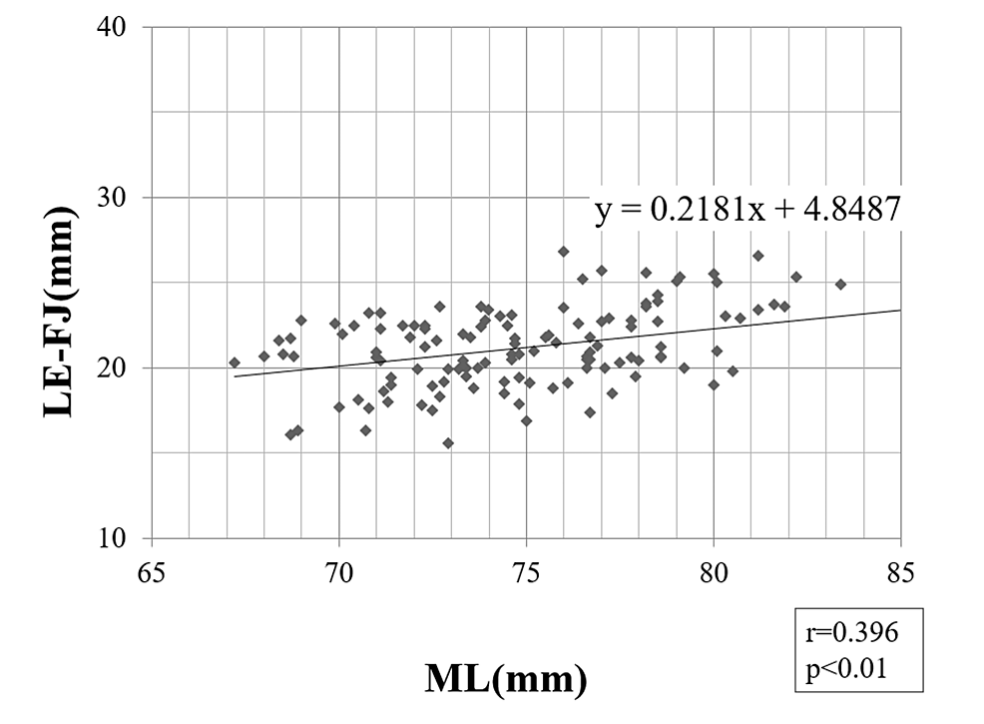
Correlation between LE-FJ and ML. LE-FJ: distance from the LE to the posterior AS in the flexed position; ML: the width of the distal femur was measured as the distance between the ME and LE; ME: medial femoral epicondyle; FJ: the JL in the flexed position; AS: articular surface; LE: lateral femoral epicondyle.

**Figure 5:**
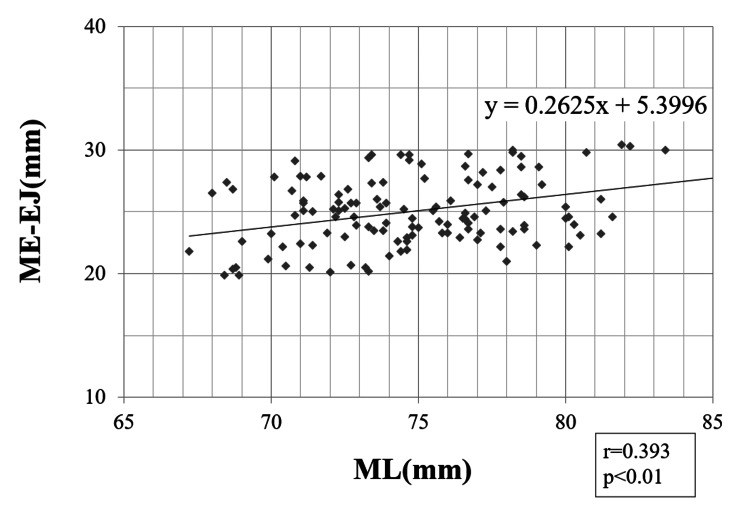
Correlation between ME-EJ and ML. ME-EJ: distance from the ME to the posterior AS in the extended position; ML: the width of the distal femur was measured as the distance between the ME and LE; ME: medial femoral epicondyle; EJ: the JL in the extended position; AS: articular surface; LE: lateral femoral epicondyle.

**Figure 6:**
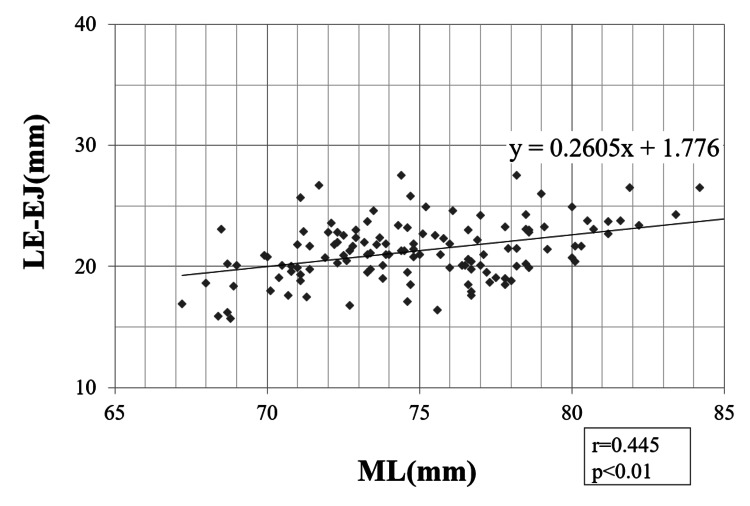
Correlation between LE-EJ and ML. LE-EJ: distance from the LE to the posterior AS in the extended position; ML: the width of the distal femur was measured as the distance between the ME and LE; ME: medial femoral epicondyle; EJ: the JL in the extended position; AS: articular surface; LE: lateral femoral epicondyle.

A strong correlation was found between the ML and ME-FJ (r = 0.623; p < 0.01) (Figure 3). In addition, we found weak-to-moderate correlations between ML and LE-FJ (r = 0.396; p < 0.01), ME-EJ (r = 0.393; p < 0.01), and LE-EJ (r = 0.445; p < 0.01) (Figures 4-6). Correlation was not observed between the ML and ME-FJ/ML, LE-FJ/ML, ME-EJ/ML, or LE-EJ/ML.

Prediction of distal femoral and posterior ASs in rTKA surgery

**Figure 7:**
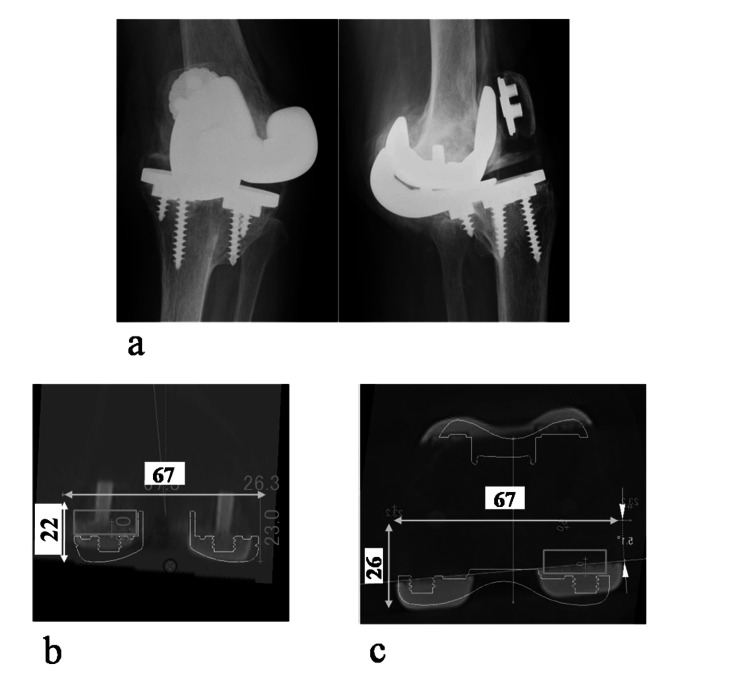
Preoperative planning for revision TKA using 3D preoperative planning software (Athena). (a) Radiographic image of revision case. (b) Coronal view: restoration of the distal femoral joint line (ML, 67 mm x 0.33 mm = 22 mm from the ME). (c) Axial view: restoration of the posterior femoral joint line (ML, 67 mm x 0.39 mm = 26 mm from the ME).

Revision arthroplasty was planned for loosening after TKA (Figure 7A). Predictive reconstruction of the AS in extension and flexion was performed using the Athena imaging software. The calculations were as follows: predicted ME-EJ - ML 67 mm (software measured) x 0.33 (obtained from the data of the present study) = 22 mm from the ME (Figure 7B); predicted ME-FJ - ML 67 mm (software measured) x 0.39 (obtained from the data of the present study) = 26 mm from the ME (Figure 7C).

## Discussion

In rTKA, the prosthetic JL tends to be malpositioned proximally [10]. Bone defects in the distal femur and posterior femoral condyle are common in rTKA; therefore, smaller prostheses are preferred to ensure direct contact with the remaining bone. Thicker inserts were employed to fill the resulting flexion gap, leading to the proximal displacement of the JL [4]. Han et al. reviewed 166 rTKAs and found that 56% of cases had JL height greater than 5 mm [9]. Buller et al. studied 327 rTKAs and found that patients with preoperative JL height within ±5 mm were 3.88 times more likely to achieve a clinical benefit in outcome scores compared to patients with JL height >5 mm (p = 0.004) [14]. Potential problems caused by elevation of the JL include patellar infra, impingement of the patellar button, accelerated wear, mid-flexion laxity, weakness of the quadriceps, anterior knee pain, and hyperextension instability [6]. Therefore, restoration of the JL is the key to a successful surgery in rTKA.

Stiehl et al. showed that the mean distance of the transepicondylar axis (TEA) to the JL was 3.08 ± 0.44 cm for the medial side and 2.53 ± 0.42 cm for the lateral side [15]. Transepicondylar width was 8.3 ± 0.98 cm in a cadaveric study of femoral condyles. There is a slight difference between these data and ours, probably due to the ethnic differences between Caucasian and Asian patients. Laskin showed that the femorotibial JL in a normal knee lies approximately 10 mm proximal to the fibular styloid and 25 mm distal to the ME of the femur [10,16]. In our study, the distance from the epicondyles to the distal and posterior JLs correlated with the width of the distal femur, and individual distances varied considerably.

Several authors have evaluated the morphology of the femoral condyles and JLs using magnetic resonance (MR) or CT imaging and found correlations between the distance from the epicondyles to the JL and the distal femoral width in Caucasians [17,18]. We compared the ratios and error values between our data and those of other studies; the results were similar despite racial differences (Table 2). The ME-FJ/ML differed depending on whether the reference point of the ME was the prominence or the sulcus [19-26]. Servien et al. recommended JL determination by using the ratio of the distance from the ME and LE to the transepicondylar width [26]. The resulting epicondyle ratio values were 0.28 (0.23-0.34) for the lateral and 0.34 (0.28-0.42) for the medial. This result is almost identical to that of our study (0.28 ± 0.03 laterally and 0.33 ± 0.04 medially). Therefore, this ratio may be used regardless of the race.

**Table 2:** Differences in ratios and values in previous literature. ME-EJ: distance from the ME to the posterior AS in the extended position; LE-EJ: distance from the LE to the posterior AS in the extended position; ME-FJ: distance from the LE to the posterior AS in the flexed position; LE-EJ: distance from the LE to the posterior AS in the flexed position; ML: the width of the distal femur was measured as the distance between the ME and LE; ME: medial femoral epicondyle; LE: lateral femoral epicondyle; EJ: the JL in the extended position; FJ: the JL in the flexed position; AS: articular surface.

References	Modality	No. of cases	ME	ME-FJ/ML	LE-FJ/ML	ME-EJ/ML	LE-EJ/ML
Tantavisut et al. [19]	MRI	140	-	-	-	0.35 ± 0.02	0.29 ± 0.02
Gao et al. [20]	X-ray	451	-	-	-	0.32 ± 0.03	0.30 ± 0.03
Hou et al. [21]	Cadaver	24	-	-	-	0.37 ± 0.05	0.28 ± 0.04
Luyckx et al. [22]	X-ray	200	-	-	-	0.32 ± 0.03	0.30 ± 0.03
Ozkurt et al. [23]	Cadaver	40	Sulcus	0.34 ± 0.02	0.29 ± 0.03	0.35 ± 0.02	0.28 ± 0.03
Lee et al. [24]	CT	50	Sulcus	0.33 ± 0.02	0.29 ± 0.02	0.31 ± 0.03	0.26 ± 0.03
Fan et al. [25]	CT	215	Prominence	0.39 ± 0.02	0.28 ± 0.03	0.33 ± 0.02	0.30 ± 0.02
Servien et al. [26]	MRI	200	Sulcus	0.34 ± 0.03	0.29 ± 0.03	0.34 ± 0.02	0.28 ± 0.02
Present study	CT	127	Prominence	0.39 ± 0.02	0.28 ± 0.03	0.33±0.04	0.28 ± 0.03

Recently, several studies have favored the use of the adductor tubercle (AT) to facilitate the determination of proper JLs [27], as it can be easily identified [28]. Di Matteo et al. reported in a systematic review that the AT ratio (distance from the AT/transepicondylar width) method can help surgeons restore the JL, achieve ligament balancing, and shorten the surgical duration in complex cases [27].

However, most of the studies cited in the systematic review [27] are based on radiographic measurements. Simple radiographs have magnification errors that make it difficult to determine the exact location of ATs. On the other hand, MRI-based studies may not accurately identify ATs in cadavers. Previous studies have shown that MRI underestimates or overestimates the length and size of measured structures [29]. Furthermore, the measured bone landmarks and the most distal apex of the AS may not be in the same imaging slice [20], resulting in measurement errors compared to measurements made on 3D samples such as 3D imaging, cadavers, and intraoperative measurements.

A positive feature of this study is that the JL was measured with reference to the cutting plane using software that could reconstruct the oblique plane. In addition, prominence was used as a landmark, which was easy to identify on CT images and during surgery. Several reports suggest that AT is a more accurate intraoperative indicator of the distal JL than the epicondyle [21,22]; however, an advantage of our method is that it allows for preoperative estimation of both distal and posterior JLs. The distance from the LE to the distal and posterior JLs was approximately 21 mm, whereas that from the ME to the distal JL and from the ME to the posterior JL was 25.2 and 29.4 mm, respectively. Thus, a difference of approximately 4 mm was observed between the distances from the ME to the distal and posterior JLs. As the sulcus of the ME lies 3-4 mm posterior to the prominence, the distance from the sulcus to the posterior JL can be assumed to be almost equal to that of the distal JL. The sulcus is the center of the insertion of the medial collateral ligament, and the distances to the distal and posterior JLs are equal. Our data support the idea that surgical TEA is a landmark for the rotational alignment of the femoral component.

Significant correlations were observed between the ML and ME-FJ, LE-FJ, ME-EJ, and LE-EJ. The values of ME-FJ/ML, LE-FJ/ML, ME-EJ/ML, and LE-EJ/ML were invariable regardless of ML, which suggested that the JL in the extended position lies at ML × 0.33 mm distal to the ME and ML × 0.28 mm distal to the LE; whereas in the flexed position, it lies at ML × 0.39 mm posterior to the ME and ML × 0.28 mm posterior to the LE. This information can be useful in determining the appropriate JL during surgery. If distal femoral width is measured during surgery and the position of the femoral component is set at ML × 0.33 mm distal and ML × 0.39 mm posterior to the ME, the appropriate JL would be restored.

Here, we determined the distance from the epicondyles to the AS; these values can be applied in rTKA. This method can also be used in the preoperative planning for rTKA, which can predict the size of the metal augmentation block, and is needed at the distal femur and posterior condyle when the AS is restored. The advantage of using the epicondyle is that it reproduces the distal femoral and posterior JLs. Therefore, the size of the femoral component and augmentation block can be planned before revision surgery.

This study had several limitations. First, only varus knees were evaluated; therefore, it is unclear whether similar results can be obtained with other deformities, such as the valgus knee. Although data from normal knees would have been desirable, it was not possible to collect CT data from healthy subjects in this study. Second, cartilage thickness was not evaluated because this study used CT images. Third, we found only a moderate correlation between the epicondyle, JL, and ML. This correlation is not high and could lead to errors in JL estimation. Fortunately, a spacing of 4 mm from the normal JL was acceptable. Under these circumstances, we believe that almost all cases can be planned within 4 mm by using our method.

However, if the epicondyles are not identifiable due to osteolysis, it may be difficult to restore the appropriate JL. In this case, AT can be used as a landmark.

Here, we used 3D digital templating software. Therefore, the measured bone landmarks and the most distal vertex of the AS could be accurately evaluated. The results of this study show that, in revision cases, the location of the femoral component, component size, need for augmentation, and size of the augmentation can be predicted in the preoperative phase rather than intraoperatively to recreate a normal articulating surface.

## Conclusions

The distance from the epicondyles to the distal and posterior JLs correlates with the distal femur width. The ratios from this study can be used to predict the location of the JL regardless of body size. These data may be useful for determining the appropriate JL for rTKA.

## References

[1] Carr AJ, Robertsson O, Graves S, Price AJ, Arden NK, Judge A, Beard DJ (2012). Knee replacement. Lancet.

[2] Kurtz SM, Ong KL, Lau E (2011). International survey of primary and revision total knee replacement. Int Orthop.

[3] Bourne RB, Chesworth BM, Davis AM, Mahomed NN, Charron KD (2010). Patient satisfaction after total knee arthroplasty: who is satisfied and who is not?. Clin Orthop Relat Res.

[4] Bellemans J (2004). Restoring the joint line in revision TKA: does it matter?. Knee.

[5] van Lieshout WA, Valkering KP, Koenraadt KL, van Etten-Jamaludin FS, Kerkhoffs GM, van Geenen RC (2019). The negative effect of joint line elevation after total knee arthroplasty on outcome. Knee Surg Sports Traumatol Arthrosc.

[6] Porteous AJ, Hassaballa MA, Newman JH (2008). Does the joint line matter in revision total knee replacement?. J Bone Joint Surg Br.

[7] Clavé A, Le Henaff G, Roger T, Maisongrosse P, Mabit C, Dubrana F (2016). Joint line level in revision total knee replacement: assessment and functional results with an average of seven years follow-up. Int Orthop.

[8] Babazadeh S, Dowsey MM, Swan JD, Stoney JD, Choong PF (2011). Joint line position correlates with function after primary total knee replacement: a randomised controlled trial comparing conventional and computer-assisted surgery. J Bone Joint Surg Br.

[9] Han HS, Yu CH, Shin N, Won S, Lee MC (2019). Femoral joint line restoration is a major determinant of postoperative range of motion in revision total knee arthroplasty. Knee Surg Sports Traumatol Arthrosc.

[10] Laskin RS (2002). Joint line position restoration during revision total knee replacement. Clin Orthop Relat Res.

[11] Mason M, Belisle A, Bonutti P, Kolisek FR, Malkani A, Masini M (2006). An accurate and reproducible method for locating the joint line during a revision total knee arthroplasty. J Arthroplasty.

[12] Hirakawa M, Miyazaki M, Ikeda S, Matsumoto Y, Kondo M, Tsumura H (2017). Evaluation of the rotational alignment of the tibial component in total knee arthroplasty: position prioritizing maximum coverage. Eur J Orthop Surg Traumatol.

[13] Akagi M, Yamashita E, Nakagawa T, Asano T, Nakamura T (2001). Relationship between frontal knee alignment and reference axes in the distal femur. Clin Orthop Relat Res.

[14] Buller LT, Metzger CM, Deckard ER, Meneghini RM (2022). The effect of joint line elevation on patient-reported outcomes after contemporary revision total knee arthroplasty. J Arthroplasty.

[15] Stiehl JB, Abbott BD (1995). Morphology of the transepicondylar axis and its application in primary and revision total knee arthroplasty. J Arthroplasty.

[16] Laskin RS (2002). Session III: total knee replacement in young patients. Clin Orthop Relat Res.

[17] Griffin FM, Math K, Scuderi GR, Insall JN, Poilvache PL (2000). Anatomy of the epicondyles of the distal femur: MRI analysis of normal knees. J Arthroplasty.

[18] Mountney J, Karamfiles R, Breidahl W, Farrugia M, Sikorski JM (2007). The position of the joint line in relation to the trans-epicondylar axis of the knee: complementary radiologic and computer-based studies. J Arthroplasty.

[19] Tantavisut S, Amarase C, Ngarmukos S, Tanavalee C, Tanavalee A (2022). Knee joint line related to bony landmarks of the knee: a radiologic study in a Thai population. Knee Surg Relat Res.

[20] Gao Z, Mao X, Xiang C, Gao Y, Zhang X, Guo Z (2021). An accurate method for locating the joint line during revision total knee arthroplasty: a radiologic study in the Chinese population. Knee.

[21] Hou Y, Jiang J, Liu H, Wang R, Wu J, Wang Y, Lin J (2023). Identification of the joint line in revision total knee arthroplasty using a multiple linear regression model: a cadaveric study. Arch Orthop Trauma Surg.

[22] Luyckx T, Beckers L, Colyn W, Vandenneucker H, Bellemans J (2014). The adductor ratio: a new tool for joint line reconstruction in revision TKA. Knee Surg Sports Traumatol Arthrosc.

[23] Ozkurt B, Sen T, Cankaya D, Kendir S, Basarır K, Tabak Y (2016). The medial and lateral epicondyle as a reliable landmark for intra-operative joint line determination in revision knee arthroplasty. Bone Joint Res.

[24] Lee M, Ho JP, Chen JY, Ng CK, Yeo SJ, Merican AM (2022). The relationship of transepicondylar width with the distal and posterior femoral condyles and its clinical implications: a three-dimensional study. J Knee Surg.

[25] Fan A, Xu T, Li X, Li L, Fan L, Yang D, Li G (2018). Using anatomical landmarks to calculate the normal joint line position in Chinese people: an observational study. J Orthop Surg Res.

[26] Servien E, Viskontas D, Giuffrè BM, Coolican MR, Parker DA (2008). Reliability of bony landmarks for restoration of the joint line in revision knee arthroplasty. Knee Surg Sports Traumatol Arthrosc.

[27] Di Matteo B, Altomare D, Dorotei A (2021). The reliability of adductor tubercle as an anatomical landmark for joint line restoration in revision knee arthroplasty: a systematic review. Ann Transl Med.

[28] Chen IH, Wu WT, Wang CC, Liu KL, Yeh KT, Peng CH (2016). An unambiguous technique for locating the adductor tubercle and using it to identify the joint line. Knee.

[29] Shaffer B, Kennedy S, Klimkiewicz J, Yao L (2000). Preoperative sizing of meniscal allografts in meniscus transplantation. Am J Sports Med.

